# Future Horizons: The Potential Role of Artificial Intelligence in Cardiology

**DOI:** 10.3390/jpm14060656

**Published:** 2024-06-19

**Authors:** Octavian Stefan Patrascanu, Dana Tutunaru, Carmina Liana Musat, Oana Maria Dragostin, Ana Fulga, Luiza Nechita, Alexandru Bogdan Ciubara, Alin Ionut Piraianu, Elena Stamate, Diana Gina Poalelungi, Ionut Dragostin, Doriana Cristea-Ene Iancu, Anamaria Ciubara, Iuliu Fulga

**Affiliations:** 1 Department of Cardiology, University Emergency Hospital of Bucharest, 169 Splaiul Independentei St, 050098 Bucharest, Romania; octav974@gmail.com (O.S.P.); elena.stamate@ugal.ro (E.S.); 2 Faculty of Medicine and Pharmacy, Dunarea de Jos University of Galati, 35 AL Cuza St, 800010 Galati, Romania; dana.tutunaru@ugal.ro (D.T.); carmina.musat@ugal.ro (C.L.M.); oana.dragostin@ugal.ro (O.M.D.); bogdan.ciubara@ugal.ro (A.B.C.); alin.piraianu@ugal.ro (A.I.P.); diana.poalelungi@ugal.ro (D.G.P.); anamaria.ciubara@ugal.ro (A.C.); iuliu.fulga@ugal.ro (I.F.); 3 Emergency County Clinical Hospital, 2 Buzaului St, 810325 Braila, Romania; ionut.dragostin@yahoo.com; 4 Saint Apostle Andrew Emergency County Clinical Hospital, 177 Brailei St, 800578 Galati, Romania; c_doriana@yahoo.com

**Keywords:** artificial intelligence, machine learning, deep learning, cardiology, electrocardiogram, echocardiography, cardiac MRI, cardiovascular imaging

## Abstract

Cardiovascular diseases (CVDs) are the leading cause of premature death and disability globally, leading to significant increases in healthcare costs and economic strains. Artificial intelligence (AI) is emerging as a crucial technology in this context, promising to have a significant impact on the management of CVDs. A wide range of methods can be used to develop effective models for medical applications, encompassing everything from predicting and diagnosing diseases to determining the most suitable treatment for individual patients. This literature review synthesizes findings from multiple studies that apply AI technologies such as machine learning algorithms and neural networks to electrocardiograms, echocardiography, coronary angiography, computed tomography, and cardiac magnetic resonance imaging. A narrative review of 127 articles identified 31 papers that were directly relevant to the research, encompassing a broad spectrum of AI applications in cardiology. These applications included AI models for ECG, echocardiography, coronary angiography, computed tomography, and cardiac MRI aimed at diagnosing various cardiovascular diseases such as coronary artery disease, hypertrophic cardiomyopathy, arrhythmias, pulmonary embolism, and valvulopathies. The papers also explored new methods for cardiovascular risk assessment, automated measurements, and optimizing treatment strategies, demonstrating the benefits of AI technologies in cardiology. In conclusion, the integration of artificial intelligence (AI) in cardiology promises substantial advancements in diagnosing and treating cardiovascular diseases.

## 1. Introduction

Cardiovascular diseases (CVDs) stand as the leading cause of early mortality and disability worldwide, with a significant rise in cases—from 271 million in 1990 to 523 million in 2019, while deaths from CVD rose from 12.1 million to 18.6 million in the same period. As a result, CVDs significantly increase healthcare costs and economic burdens [1,2]. Addressing this growing challenge requires cost-effective, scalable interventions, with artificial intelligence (AI) poised to play a pivotal role [3].

The definition of artificial intelligence (AI) can be summarized as the use of a computer or a technology to simulate cognitive skills that are similar to human beings, such as critical thinking and intelligent behavior [4]. This term was first introduced by John McCarthy in 1956 as “the science and engineering of making intelligent machines” [4,5]. By replicating human cognitive functions, AI has transformed industries, enhanced efficiency, and opened up new possibilities [6]. Nowadays, AI has been rapidly developed and implemented in many routine clinical care fields, such as enhanced prognosis and diagnosis, surgeries assisted by robots, rehabilitation, and precision medicine. The decision-making process for a patient requires professionals able to perform history collection, diagnosis, and treatment strategies. Delaying the decision-making process is a consequence of the large amount of work needed for the proper care of patients. Accurate and reproducible approaches with the help of AI, combining human knowledge enhanced with technology power, can solve this problem [7]. For example, this can be achieved by eliminating repetitive tasks that are time-consuming and helping the physician focus on human-to-human bonding, application of judgment, and emotional intelligence [8].

**Figure 1:**
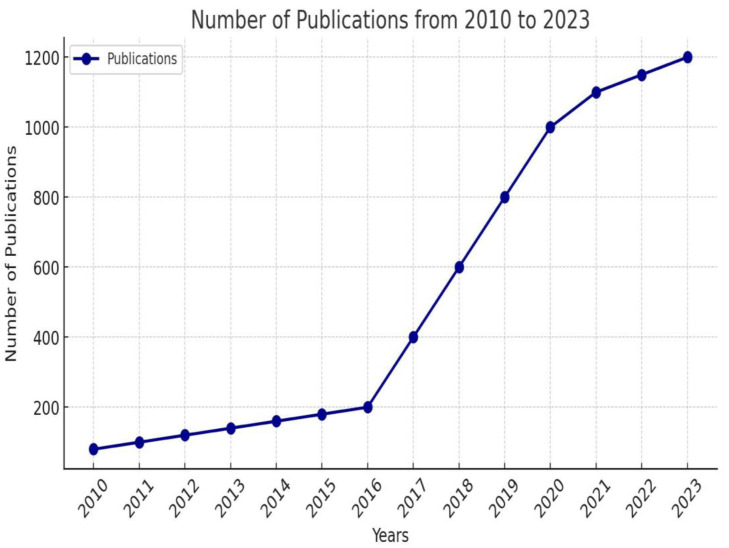
Number of publications related to artificial intelligence in cardiology from 2010 to 2023—adapted from [9].

Zhang J. et al. highlighted the growing interest in artificial intelligence research in cardiology. They studied the Web of Science database, finding 4611 articles published between the years 2000 and 2023 on this topic. As shown in Figure 1, the number of articles has significantly risen over the past 13 years, from 2010 to 2023. They predict that in the coming years, the interest in this area of publication will continue to rise [9].

### 1.1. Terminology

According to Nick Bostrom, AI has three major levels of development. Artificial Narrow Intelligence (ANI) is an algorithm that can perform only a single task which is to recognize patterns in huge data sets. It can easily solve classifications based on texts, voices, or images. Artificial General Intelligence (AGI) is designed to match human-level intelligence in terms of comprehensibility and total cognitive capacity. Artificial Superintelligence (ASI) is a term used for an AI that can exceed human capabilities [10,11].

#### 1.1.1. Machine Learning (ML)

ML represents a component of artificial intelligence (AI) that covers two primary predictive methodologies: supervised learning and unsupervised learning. Supervised learning means that datasets feature known labels such as prediction targets, whereas in unsupervised learning, datasets lack such labels [12]. Reinforcement learning (RL) is one of the least utilized types of machine learning in healthcare. This is due to its inherent complexity, unpredictability, and lack of explainability. In RL, algorithms are trained to make a series of decisions within a highly complex environment, aiming to achieve a defined goal [13,14]. There are three key elements essential for effective ML implementation: a reliable dataset, an ML algorithm, and an unbiased model evaluation. A reliable dataset may be structured or unstructured and should contain minimal noise, outliers, and missing values [12].

#### 1.1.2. Deep Learning (DL)

**Figure 2:**
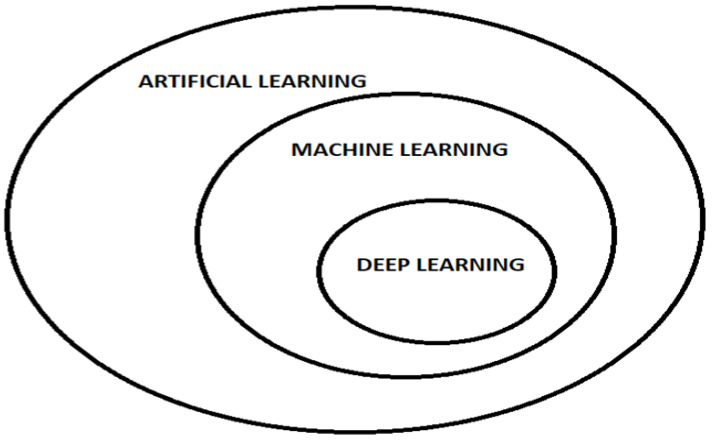
Relationship between AI, ML, and DL—adaptation from [15].

Deep learning (DL) is a subset of machine learning (see Figure 2) that uses computational models and algorithms to replicate the architecture of biological neural networks in the brain, known as artificial neural networks (ANNs). When the brain processes new information, it compares it to known information to interpret it. It categorizes and labels the information, a method also employed by DL to decipher and categorize data [15]. DL is gaining significant attention from researchers in the medical and healthcare fields as it has the potential to enhance the precision of various medical applications by using medical data [16]. It is particularly effective for processing unstructured data such as speech, images, or text. However, it typically does not offer insights into which aspects of the data are influencing these functions [13].

#### 1.1.3. Artificial Neural Networks (ANNs)

Artificial neural networks (ANNs), simply neural networks (NNs), consist of interconnected units known as neurons, nodes, or neural units. These networks are inspired by the human brain, utilizing a plethora of terms from neuroscience. For a system to qualify as an artificial neural network, it must have a labeled directed graph where the nodes execute basic computations. In these networks, nodes perform simple operations, and each connection transmits a signal from one node to another, with a number known as the “Connection Strength” or “Weight” that modulates the signal’s intensity. Neural networks are widely applied in various areas such as signal processing, pattern recognition, speech processing, handwriting recognition, time series prediction, data compression, feature extraction, and general pattern recognition. These systems serve as alternatives to human expertise and knowledge, offering the advantage of being relatively straightforward to tailor to specific problems and their capability for processing nonlinear data [17,18].

#### 1.1.4. Convolutional Neural Networks (CNNs)

Convolutional neural networks (CNNs) are another type of neural network that, similar to traditional neural networks, consists of neurons with learnable weights. However, CNNs are specifically designed for inputs, such as images, that have inherent structures. This assumption is incorporated into their architecture by using shared weights across different locations in an image and designing neurons to respond only to local areas [19]. In fields like automatic diagnostics, CNNs are commonly utilized for tasks like image classifications. They have demonstrated higher accuracy and efficiency compared to traditional classification methods [20,21].

## 2. Materials and Methods

The present paper reviews the most recent potential clinical applications of AI in the field of cardiology, specifically using paraclinical investigations. We conducted a review of English language research articles in this field published only in the last 2 years (2023, 2024). This approach highlights recent advancements and addresses contemporary challenges and trends. Additionally, it provides a forward-looking perspective on emerging innovations and practices. This focus ensures the review remains highly relevant and informative. Studies were retrieved from the Science Direct, PubMed, and Google Scholar databases using combinations of the following keywords: “artificial intelligence”, “deep learning”, “machine learning”, “cardiology”, “electrocardiogram”, “echocardiography”, “coronary angiography”, “cardiac computed angiography”, and “cardiac MRI”. The relevant search results were manually selected, resulting in more than 100 relevant manuscripts. The inclusion criteria focused on the application of artificial intelligence in cardiac paraclinical investigations, future perspectives, and performance metrics like sensitivity, specificity, accuracy, precision, or correlation between automated and manual measurements. The exclusion criteria were the language of publication, date of publication, and studies that do not directly assess or utilize AI technologies in cardiology.

## 3. Results

Following a thorough examination and evaluation of 127 articles, we selected 31 papers that were particularly pertinent to our study. This selection includes 11 studies focused on electrocardiography (ECG), 14 on echocardiography, 1 on coronary angiography, 2 on cardiac computed angiography, 1 on computed tomography, and 2 on cardiac MRI, as detailed in Table 1. These studies offered significant insights into the application and impact of AI in various paraclinical tests commonly used in cardiology, thus establishing the foundation of our review.

### 3.1. Electrocardiography (ECG)

An electrocardiogram (ECG) captures the heart’s electrical activity. It serves as a non-invasive tool for several biomedical uses, including monitoring heart rate, assessing heartbeat rhythm, and detecting cardiac abnormalities. However, the interpretation of an ECG requires deep knowledge [53]. Artificial intelligence can help create diagnostic models, streamlining the process and reducing the reliance on the observer’s expertise.

Herman R. et al. created an ECG AI model used for detecting acute coronary occlusion myocardial infarction (OMI) which has a higher accuracy than classical STEMI criteria. This indicates its ability to boost ACS triage, ensuring the timely and proper referral for immediate vascularization [22].

We found a paper written in 2024 that focused on validating an ECG-derived marker for pediatric Heart Failure (HF) that predicts the risk of future cardiovascular events. ECG-based convolutional neural networks that detect neurohormonal activation could serve as a valuable indicator of pediatric HF, offering a more distinct prognostic potential than HF [23].

Electrocardiographic left ventricular hypertrophy with a strain pattern serves as a marker for left ventricular hypertrophy [54]. Several clinical conditions can cause the development of LVH, including essential hypertension, athletic heart with physiological LVH, hypertrophic cardiomyopathy without or with outflow tract obstruction (HCM, HOCM), and infiltrative cardiac processes (e.g., Amyloidosis, Fabry disease, Danon disease) [55]. Hilis J. et al. examined the accuracy of an AI device, Viz HCM, for ECG screening of hypertrophic cardiomyopathy. The device identified HCM based on a 12-lead ECG. It has the potential to be used as a screening tool to augment current care pathways. It has received approval from the US Food and Drug Administration [24]. Haimovich J. et al. developed an artificial intelligence model based on a convolutional neural network that can detect and classify causes of LVH patterns on an ECG, such as cardiac amyloidosis, hypertrophic cardiomyopathy, aortic stenosis, hypertensive LVH, and has better results than clinical ECG-based rules [25]. A new AI-ECG model designed to identify cardiac amyloidosis showed strong overall performance. However, reduced effectiveness was observed in patients with left bundle branch block, left ventricular hypertrophy, and within ethnically diverse groups, underscoring the need for targeted validation in these subgroups [26].

Heart Failure (HF) is a complex, life-threatening condition associated with high morbidity and mortality, reduced quality of life and functional capacity, and substantial healthcare costs. Affecting over 64 million people globally, HF is recognized as a worldwide pandemic [56]. Butler L. et al. developed AI models based on ECG data that predict the risk of Heart Failure (HF) within a 10-year period with accuracy comparable to or better than current HF risk calculators and traditional ECG methods. This research has several objectives, including the potential use of these AI models to enable cost-effective and remote monitoring of at-risk groups, thereby facilitating prompt interventions and enhancing clinical decision-making [27].

Atherosclerotic cardiovascular disease (ASCVD), with coronary artery disease (CAD) as its primary driver, is the leading cause of death globally. While ASCVD risk estimators like the pooled cohort equations (PCE) are used for risk stratification and primary prevention, their accuracy remains less than ideal. ECG-AI models have been developed to identify specific conditions: (1) elevated coronary artery calcium (CAC), (2) obstructive CAD, and (3) regional left ventricular (LV) akinesis as an indicator of a potential previous myocardial infarction (MI). These models provide complementary insights and identify distinct risk profiles. They may offer a solution to the gaps in existing risk stratification methods, especially for patients where PCE does not accurately estimate ASCVD risk, or when assessments over periods shorter than 10 years are necessary [28]. Lee Y. et al. conducted a study on an AI-enabled model that integrates deep learning and machine learning to help identify patients with obstructive coronary artery disease (CAD). This model achieves diagnostic performance comparable to traditional methods based on cardiovascular risk factors (CVRFs). It could prove to be a valuable clinical tool in outpatient settings to determine which patients might require further diagnostic testing for a stable CAD [29].

Pulmonary embolism (PE) is a potentially fatal condition characterized by diagnostic challenges due to its non-specific clinical symptoms. Confirmation of PE requires computed tomography pulmonary angiography (CTPA). An AI model designed using 12-lead ECG data was created by Valente Silva B. et al. and showed exceptional specificity (100%) in detecting PE, surpassing the accuracy of standard clinical prediction rules [30].

The electrocardiogram (ECG) is a crucial non-invasive instrument used in cardiology to diagnose arrhythmias [57]. A convolutional neural network has been developed to distinguish between AVRT and AVNRT. The accuracy of this neural network is currently moderate, but could potentially increase with a larger dataset. This model was initially trained using data from just 124 patients undergoing EP studies. Accurately diagnosing the mechanism of arrhythmia using a 12-lead ECG can enhance preprocedural counseling, consent processes, and planning of the procedure [31]. Shimojo M. et al. created a machine learning algorithm to differentiate between the left ventricular outflow tract (LVOT) and the right ventricular outflow tract (RVOT) origins of outflow tract ventricular arrhythmias (OTVA). This algorithm is based on four key elements: the aVF/II R-wave ratio, the V2S/V3R index, the QRS amplitude in lead V3, and the R-wave deflection slope in lead V3. It has demonstrated high accuracy and could be useful in planning catheter ablation procedures for OTVA [32].

#### 3.1.1. Echocardiography

Echocardiography has seen tremendous and exponential growth over the decades, becoming an essential tool for cardiac assessments. Originally beginning with the B-mode, the field has evolved with advancements in technology such as Doppler and three-dimensional imaging. This has made echocardiographic examinations more detailed and comprehensive. Echocardiography has several uses, such as:

- Assessing and monitoring the left ventricular systolic and diastolic function;
- Evaluation of the right ventricular function;
- Evaluation and quantification of the cardiac chamber size;
- Assessing the functional significance of a valvular lesion and the evaluation of the prosthetic valve structure and function;
- Identification of the cardiac source of embolism and the evaluation of the cardiac masses;
- Evaluation of pericardial diseases [58].

Artificial intelligence (AI) can assist healthcare providers by serving as a valuable diagnostic tool in the field of echocardiography, especially in automatic measurements and interpreting the results. Furthermore, AI can enhance research capabilities and reveal new approaches in medical management [59].

A study confirmed the efficacy of an AI model created to perform automatic measurements on transthoracic echocardiography. It was trained using over 3000 TTE scans from both healthy individuals and heart disease patients. The key outcomes included high AI accuracy compared with manual measurements and shorter examination time. Also, it showed lower interobserver variability, highlighting its potential as a training tool [33]. Sveric K. et al. compared an AI-based automated workflow that merges clip selection and LVEF calculations with a modified biplane Simpson (MBS) technique, using cardiac magnetic resonance imaging (CMR) as the standard reference. The AI demonstrated greater accuracy and reliability compared to the MBS method, reducing user-related variability and, therefore, the potential to improve the clinical utility of echocardiography. This algorithm has gained approval from the Food and Drug Administration (FDA) and Conformité Européenne (CE) [34]. Another study was published about a deep learning model that was developed to detect region wall motion abnormalities. Its results are similar to experts and surpassed many novices. This model may improve the efficiency of RWMA assessment and serve as a teaching tool for novices [35].

A number of articles focus on AI models which can assess right ventricle parameters. The first study investigates an attention-based deep learning (DL) approach for quantifying the right ventricle in 2D echocardiography, demonstrating both feasibility and promising accuracy. This model was evaluated against cardiac magnetic resonance imaging as the reference standard [36]. Murayama M. et al. created a fully automated deep learning (DL) tool designed to estimate right ventricular ejection fraction (RVEF) from two-dimensional echocardiographic videos of apical four-chamber views in patients with precapillary pulmonary hypertension (PH). The study demonstrated that this tool can accurately estimate RVEF and match the diagnostic performance of human readers in detecting right ventricular systolic dysfunction. Cardiac magnetic resonance (CMR) was also used as the standard reference in this research [37]. A new artificial intelligence (AI) software, LVivoRV, was evaluated for its precision in measuring right ventricular (RV) parameters, such as fractional area change (FAC), free wall strain (FWS), and tricuspid annular planar systolic excursion (TAPSE). The software displayed excellent sensitivity and a negative predictive value for detecting RV dysfunction, although it showed low specificity and positive predictive value. Nonetheless, its ability to reliably exclude significant RV dysfunction should be emphasized [38]. Recently, a machine learning model trained on data from patients who underwent right-sided heart catheterization and transthoracic echocardiography was used to predict pulmonary hypertension (PH) through echocardiography. The model demonstrated high sensitivity and positive predictive value, although its negative predictive value was lower. This machine learning method shows potential for accurately identifying patients unlikely to have PH [39].

In developed countries, aortic stenosis is the most common form of valvular heart disease. This condition typically manifests as part of the aging process and is becoming increasingly common as the population’s average age rises. Without treatment, severe symptomatic aortic stenosis is universally fatal [60]. Echocardiography plays a crucial role in diagnosing, assessing, and managing aortic valve disease in patients [61]. The Digital Aortic Stenosis Severity Index (DASSi) is a video-based AI tool that is capable of detecting severe aortic stenosis (AS) using single-view long-axis echocardiography, without requiring Doppler characterization. A recent study has demonstrated that DASSi can independently predict the development and progression of AS. This enables opportunistic risk stratification across various cardiovascular imaging modalities and suggests the potential for use on handheld devices [40]. Krishna H. et al. conducted a study comparing the measurement accuracy of an artificial neural network (Us2.ai) with that of trained echocardiographers in assessing aortic stenosis. The echocardiographic images analyzed included patients with normal aortic valves and those with varying degrees of aortic stenosis. Their findings suggest that Us2.ai is capable of closely matching human measurements of all critical parameters used in evaluating the severity of AS. This could potentially decrease interscan variability, enhance the interpretation and diagnosis of AS, and allow precise and reproducible management of patients with AS [41].

A machine learning algorithm was developed using echocardiographic features from patients undergoing coronary angiography. The model displayed high sensitivity (0.952) but lower specificity (0.691), indicating its strong capability to detect coronary artery disease (CAD) but also suggesting a potential for a high rate of false positives. Further analysis revealed that false-positive cases were more prone to cardiac events compared to true-negative cases. The model may be valuable in assessing the likelihood of CAD before tests and for screening patients in clinical settings [42]. Another study aimed to assess the accuracy of a machine learning (ML) model, specifically an eXtreme Gradient Boosting (XGBoost) model, in predicting five-year all-cause mortality among patients with chronic coronary syndromes (CCS) using clinical and transthoracic echocardiography (TTE) data, with the key risk predictor including LV dysfunction and significant tricuspid regurgitation. The performance of the XGBoost model was evaluated against traditional risk stratification scores. The ML model outperformed the traditional risk scores. This highlights the potential of using advanced ML models to enhance risk prediction in CCS patients beyond traditional methods [43].

We have found three more articles related to echocardiography, which can bring new perspectives for the future. Atrial fibrillation (AF) is one of the most common arrhythmias in adults, with an estimated prevalence between 2% to 4% [62]. Lu N. et al. proposed an artificial intelligence (AI) model designed to detect atrial fibrillation (AF) using apical four-chamber (AP4) cines, independently of the electrocardiogram (ECG) data. The performance assessment used a cardiologist’s evaluation of the ECG rhythm strip as the gold standard. The results indicate that this AI model can accurately detect AF using echocardiography, achieving the accuracy of a cardiologist’s analysis based on ECG [44]. Another study highlights an ML model designed to detect rheumatic heart disease by analyzing the mitral valve. It emphasizes the model’s capability to identify RHD with an accuracy comparable to that of expert cardiologists [45]. Steffner K. et al. pioneered the AI-based interpretation of TEE images by developing a convolutional neural network (CNN) to accurately identify standardized TEE views. This demonstration of effective AI classification opens the door to advanced deep learning analyses in intraoperative and intraprocedural TEE imaging [46].

#### 3.1.2. Coronary Angiography

Percutaneous coronary intervention (PCI) is the standard treatment for ischemic heart disease. Precise evaluation of stenosis and the reference diameter is essential. Since visual assessments of lumen diameter and stenosis lack objectivity, quantitative coronary angiography (QCA) is commonly used in clinical settings to evaluate coronary artery stenosis [63]. A study published in 2024 introduced a new approach for artificial intelligence-based quantitative coronary angiography (AI-QCA) to analyze major vessels. AI-QCA was created using three deep learning models trained on angiographic data to accurately delineate lumen boundaries. The model showed good sensitivity in detecting lesions and had strong correlations with manual QCA. AI-QCA successfully identified and quantitatively analyzed multiple lesions in major vessels. This shows this model’s potential as an automated solution in coronary angiography, providing significant benefits for the quantitative evaluation of coronary lesions and supporting clinical decision-making [47].

#### 3.1.3. Cardiac Computed Angiography

Coronary computed tomography angiography (CCTA) plays an essential role in assessing coronary artery disease (CAD). This non-invasive method is especially valuable for patients with a low to intermediate risk of ischemic heart disease, highlighting its importance in evaluating stable patients who do not need immediate revascularization [64]. AI-QCPA (HeartFlow) employs a CCTA-derived 3D model specific to each patient, illustrating the arterial lumen and outer wall to quantify and characterize plaque. A recent paper by Rinehart S. et al. indicates that incorporating AI-QCPA into routine CCTA reports or interpretations can modify medical management recommendations in 66% of cases. This significant influence is primarily due to the fact that most patients with symptoms indicative of CAD, who show any coronary atherosclerosis on CCTA, are advised to begin aspirin and high-intensity statin therapies. The inclusion of AI-QCPA data leads to more aggressive and elevated medical treatment strategies [48]. A different study assessed the effectiveness of AI-QCT in identifying low-density non-calcified plaque (LD-NCP) compared with a near-infrared spectroscopy-intravascular ultrasound. AI-QCT displayed a high diagnostic accuracy in identifying significant LD-NCP. Moreover, the measurements of the vessel area, lumen area, plaque burden, and lesion length from AI-QCT closely matched the corresponding measurements from the intravascular ultrasound (IVUS) [49].

#### 3.1.4. Computed Tomography

Computed tomography (CT) provides a unique capability for the simultaneous evaluation of the aortic root and vascular anatomy, making it the gold standard for patients being evaluated by transcatheter aortic valve replacement (TAVR). This comprehensive approach provides critical information for determining patient eligibility, selecting the appropriate prosthesis size, and determining the optimal access strategy [65]. Toggweiler S. et al. created an artificial intelligence (AI)-powered software(4TAVR) designed to automatically generate anatomical measurements and other data for TAVR planning and implementation. The annular measurements produced by the AI demonstrated excellent concordance with those taken manually by expert operators. This software not only saves time but also enhances efficiency and reduces costs. The authors concluded that it may be especially beneficial for high-volume centers burdened by the extensive analysis of CT scans and for low-volume centers with limited experience in such analyses [50].

#### 3.1.5. Cardiac MRI

Cardiovascular magnetic resonance (CMR) encompasses several magnetic resonance imaging (MRI) techniques developed to analyze cardiovascular morphology, ventricular function, myocardial perfusion, tissue characterization, flow quantification, and coronary artery disease [66]. A study released this year evaluated an automatic tool designed to measure cardiac volumes on CMR to evaluate its clinical feasibility. The tool’s automatic segmentation of both ventricles on CMR showed excellent concordance with manual segmentation performed by CMR experts in a retrospective clinical cohort. The implementation of this tool could enhance the efficiency of CMR reporting and minimize delays between imaging and diagnosis [51]. Scar burden determined by late gadolinium enhancement (LGE) CMR is predictive of all-cause mortality. In patients with coronary artery disease, the mass of myocardial scars can be accurately determined using LGE cardiac MRI. Ghanbari F. et al. tested a machine learning model for the quantification of myocardial scar mass, which demonstrated excellent accuracy. This method could potentially improve the current prediction models that use guideline-based risk criteria for implantable cardioverter defibrillator implantation [52].

## 4. Discussions

Artificial intelligence can be applied in many directions in cardiology and in medicine. However, the studies reviewed share similar limitations, such as limited validation and currently restricted applicability. Nevertheless, the future prospects seem excellent, as long as there is a resolution to the problems of implementing artificial intelligence on a large scale in medicine.

### 4.1. Challenges in AI Implementation

He J. et al. identify several critical issues in implementing AI technologies in medicine, such as the need for data sharing, ensuring transparency, guaranteeing patient safety, and standardizing data for seamless integration into existing clinical workflows. They also discuss the financial aspects of AI implementation and the importance of educating the medical workforce. It also underlined the necessity to develop regulatory standards to assess the safety and efficacy of AI technologies. The Data Science Institute, founded by the American College of Radiology, is noted as an example of an initiative supporting AI implementation [67]. Kella L. et al. point out that “AI algorithms are only as good as the data and assumptions they are fed”. Ensuring diverse, representative data in the training, evaluation, and post-deployment monitoring phases is critical. It is crucial to use diverse and representative data throughout the training, evaluation, and ongoing monitoring phases to prevent biases and overfitting, which can lead to inaccurate AI predictions in real-world applications. They also note that human biases can influence AI’s clinical use. A survey revealed that while many Korean physicians recognized AI’s usefulness in diagnosis, few were well-versed in AI, and some doubted AI’s effectiveness in unforeseen circumstances [68,69].

A significant challenge for AI tools in healthcare is skepticism due to the opaque nature of many algorithms, often referred to as “black box” systems [70]. These “black box” methods, including neural networks, random forests, and gradient boosting models, can make it difficult for physicians and researchers to understand or explain specific diagnostic or treatment recommendations [71,72]. In contrast, “white box” algorithms like logistic regression and decision trees are more transparent [71]. Skepticism is not just among clinicians, patients often show even less tolerance for errors made by AI than by human clinicians [73]. However, Duran J. et al. state that the use of medical AI in routine clinical practices can be significantly advantageous even when relying heavily on less transparent “black box” algorithms. They believe that the issues and challenges that arise in this setting should shape the further development of these technologies and the education of medical professionals. This approach aims to prepare healthcare workers to effectively incorporate these complex systems into standard medical practice [74].

Gerkes S et al. identified four primary ethical issues (one regarding informed consent, one regarding safety and transparency, one regarding biases, and one about data privacy) and five legal issues such as safeness and effectiveness, liability, data protection and privacy, cybersecurity as well as intellectual property law [75]. Tang L. et al. conducted a study revealing concerns among patients and clinicians about medical AI’s potential to reduce human interactions and trust in healthcare communication. Physicians highlighted autonomy and justice as critical ethical concerns in using medical AI. Clinicians also raised issues regarding equity, discrimination, stigma, and distributive justice. Additionally, the publishers pointed out the lack of research into AI developers’ understanding of ethics other than their research to fix AI biases, connected to the ethical principles of fairness and justice [76].

There is a crucial need to standardize the metrics used in AI-based research as currently the results are reported using varied performance metrics like accuracy, sensitivity, and specificity, making them hard to compare. Additionally, while models may perform exceptionally in theoretical or in silico studies, translating these successes to clinical settings often proves challenging [77,78].

### 4.2. Limitations

Our literature review may encounter several limitations. These include potential selection bias, as the review is restricted to studies published in English or to those accessible in particular journals, possibly overlooking significant research. Variability in the quality and methodologies of the included studies may also affect the reliability of conclusions drawn. Additionally, the rapid evolution of the field might mean that the review could miss some of the most recent developments in AI applications in cardiology.

## 5. Conclusions

The integration of artificial intelligence (AI) in cardiology opens exciting possibilities that have the potential to revolutionize the field through the strategic use of big data, machine learning algorithms, and neural networks. This innovation promises improved processes in triage, diagnosis, prognosis, monitoring, and treatment of cardiovascular diseases. Numerous studies have underlined the potential use of AI in paraclinical investigations, such as ECG, echocardiography, coronary angiography, computed tomography, and cardiac MRI.

Despite the enthusiasm surrounding AI in cardiology, several challenges must be addressed to maximize its potential. It is crucial to standardize AI algorithms and the metrics used to evaluate them to ensure uniformity across research and clinical usage. Therefore, the judicious utilization of AI technologies, guided by evidence-based practices and ethical principles holds the key to unlocking the full potential of artificial intelligence in advancing medical care and improving patient outcomes.

While AI in cardiac paraclinical investigations is still in its early age, its potential to transform the field is immense. Essentially, AI is not a replacement for human expertise. Instead, it serves as a valuable collaborator. Human discernment and knowledge remain indispensable for interpreting context and making informed decisions. By addressing the current challenges and fostering a collaborative, informed approach to its development and use, AI can significantly enhance cardiac care, making it more precise, efficient, and patient-centered.

## Figures and Tables

**Table 1:** Summary of the recent AI studies in cardiology.

Paraclinical Investigation	Author	Year of Study	Application
**ECG**	Herman R. [22]	2024	Detection of occlusion myocardial infarction.
	Nogimori Y. [23]	2024	ECG-derived CNN is a novel marker of HF in children with different prognostic potential from BNP.
	Hillis J. [24]	2024	Identification of hypertrophic cardiomyopathy on a 12 lead ECG. Classification of hypertrophic cardiomyopathy, cardiac amyloidosis, and echocardiographic LVH. Detection of cardiac amyloidosis.
	Haimovich J. [25]	2023	
	Harmon D. [26]	2023	
	Butler L. [27]	2023	Early Heart Failure prediction using ECG-AI models.
	Awasthi S. [28]	2023	Assessing the risk stratification of CAD.
	Lee Y. [29]	2023	
	Valente Silva B. [30]	2023	Diagnosis of Acute Pulmonary Embolism.
	Sau A. [31]	2023	Distinguish AVRT from AVNRT.
	Shimojo M. [32]	2024	Identification of the origin of outflow tract ventricular arrhythmia.
**Echocardiography**	Shiokawa N [33]	2024	Automatic measurements of transthoracic echocardiography.
	Sveric K. [34]	2024	Calculation of left ventricular ejection fraction.
	Slivnick J. [35]	2024	Detection of Regional Wall Motion Abnormalities.
	Kampaktsis P. [36]	2024	Quantification of the right ventricle.
	Murayama M [37]	2024	Measuring the right ventricle ejection fraction.
	Hsia B. [38]	2023	Assessing the parameters of right ventricular dysfunction.
	Anand V. [39]	2024	Diagnosis of pulmonary hypertension.
	Oikonomu E. [40]	2024	A video-based biomarker for detection of severe aortic stenosis.
	Krinsha H. [41]	2023	Assessment of aortic stenosis.
	Guo Y. [42]	2023	Detection of coronary artery disease.
	Molenaar M. [43]	2024	Identifying high-risk chronic coronary syndrome patients.
	Lu N. [44]	2024	Detection of atrial fibrillation on echocardiography without ECG.
	Brown K. [45]	2024	Detecting rheumatic heart disease.
	Steffner K. [46]	2024	Identification of standardized Transesophageal Echocardiography views.
**Coronary Angiography**	In Kim Y. [47]	2024	Quantitative assessment of coronary lesions.
**Cardiac Computed Angiography**	Rinehart S. [48]	2024	Plaque quantification.
	Omori H. [49]	2023	Morphology of coronary plaque.
**Computed Tomography**	Toggweiler S. [50]	2024	Planning of transcatheter aortic valve replacements.
**Cardiac MRI**	Salehi M. [51]	2024	Automated segmentation of both ventricles on CMR by an automatic tool.
	Ghanbari F. [52]	2023	Prediction of major arrhythmic events by analyzing cardiac MRI scar.

## Data Availability

Not applicable.
